# Conservative Management on Antenatally Found Congenital Cystic Adenomatoid Malformation: A 1‐Year Follow‐Up Case Report

**DOI:** 10.1155/crog/2170565

**Published:** 2026-07-18

**Authors:** Bambang Abimanyu, Muhammad Robyanoor Ahyadi Radaam, Ruth Widhiati Raharjo Putri, Yosef Dwi Cahyadi Salan, Hermin Sabarudin, Adek Yeary Wardhani, Yakob Togar

**Affiliations:** ^1^ Faculty of Medicine and Health Sciences, Ulin Regional Hospital, Lambung Mangkurat University, Banjarmasin, Indonesia, unlam.ac.id

**Keywords:** case report, CCAM, prenatal diagnosis

## Abstract

**Objective:**

We report a rare case of congenital cystic adenomatoid malformation (CCAM) diagnosed antenatally based on USG and postnatally based on babygram, CT scan, and chest x‐ray. We follow up the baby up to 1 year of age.

**Method:**

This study is a retrospective case report describing the clinical presentation, diagnosis, and management of a single patient.

**Results:**

The patient is an outpatient clinic with a diagnosis of G2P1A0, gestational age 38–39 weeks with breech presentation, not in labor, and the fetus is suspected for CCAM. The patient was planned for termination with cesarean delivery. The patient was first diagnosed with CCAM based on antenatal USG examination: A cystic mass in the thorax region of the fetus measuring 1.5 cm was found. The baby was then born by CS in consideration of breech presentation. At birth, the baby had a good APGAR score of 7‐8‐9. The baby underwent a babygram at 2 days old, which showed a CPAM type III result; CT scan at 16 days old showed infected CPAM type I/CCAM. The baby has been observed for 1 year of life, and no signs of respiratory distress were found. The latest chest x‐ray at 1 year of age showed left superior pulmonary cyst.

**Conclusion:**

CCAM can be diagnosed perinatally by ultrasound examination. Management of CCAM depends on the size of the lesion and whether significant respiratory distress is present in the newborn. In our case, the CCAM was established prenatally and postnatally; no signs of respiratory symptoms were found within 1 year of age.

## 1. Introduction

Congenital cystic adenomatoid malformation (CCAM) is a congenital airway malformation, a developmental, nonhereditary, and hamartomatous lung disorder of unknown etiology with an incidence of 1 in 25,000 to 1 in 35,000 pregnancies [1, 2].

During antenatal period, routine ultrasound examination with Doppler and MRI of the fetus will assist in the accuracy of CCAM diagnosis. USG and MRI are used in identifying the location of lung abnormalities and characteristics [3]. Postnatal management of CCAM depends on whether the patient is having respiratory distress or asymptomatic. In symptomatic patients, CCAM is managed with surgical resection. The surgery is curative and has few complications [4].

We report a very rare case of CCAM. This case was discovered when the patient underwent antenatal ultrasound with the discovery of a cystic part in the right lung. The patient had a good outcome during the follow‐up period until 1 year of age.

## 2. Case Report

**Figure 1:**
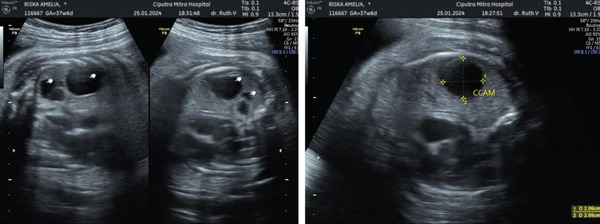
Initial ultrasound of suspected CCAM.

Mrs. R, a 25‐year‐old patient, is from an outpatient clinic with a diagnosis of gravida 2 with a breech presentation not in labor and a fetus with CCAM abnormalities. The patient was planned to be terminated with CS and planned for neonatology evaluation. This pregnancy is the second pregnancy with unremarkable previous pregnancies. The patient has no history of hypertension, diabetes, or other diseases; from the familial history of the disease, there were no congenital abnormalities. The patient was first diagnosed with suspected CCAM in the fetus during an antenatal ultrasound examination at 38 weeks of gestation. The results of the ultrasound examination showed a suspicion of cystic mass in the right fetal hemithorax: mass width of 2.06 cm, height of 4.00 cm, length of 2.06 cm, head circumference of 33.77 cm, andcongenital pulmonary airway malformation volume = 8.83 cm^3^/congenital pulmonary airway malformation volume ratio (CVR) = 0.26 cm^2^ (Figure 1). No follow up was made regarding change of lesion size as it was diagnosed at term and she had planned for termination of pregnancy. The timeline summary of the patient′s case is presented (Table 1).

**Table 1:** Timeline of CCAM case.

Timepoint	Clinical event
Antenatal period	Routine obstetric ultrasound detected a cystic lesion in the fetal right hemithorax CVR was 0.26, with no fetal hydrops reported.
38–39 weeks of gestation	Mother was admitted with breech presentation and suspected fetal CCAM, cesarean delivery was planned.
Birth (Day 0)	Male infant was delivered by cesarean section, birth weight 3075 g, with Apgar scores of 7‐8‐9 and no immediate respiratory distress.
Postnatal day 2	Babygram/chest radiograph suggested CPAM type III, pediatric surgery consultation was obtained.
Postnatal day 16	Chest CT showed a right intrapulmonary cystic lesion with air–fluid level, impression: infected CPAM type I/CCAM.
Age: 1 year	Clinical follow‐up showed no respiratory distress, latest chest x‐ray demonstrated a persistent left superior pulmonary cyst, and conservative follow‐up was continued.

**Figure 2:**
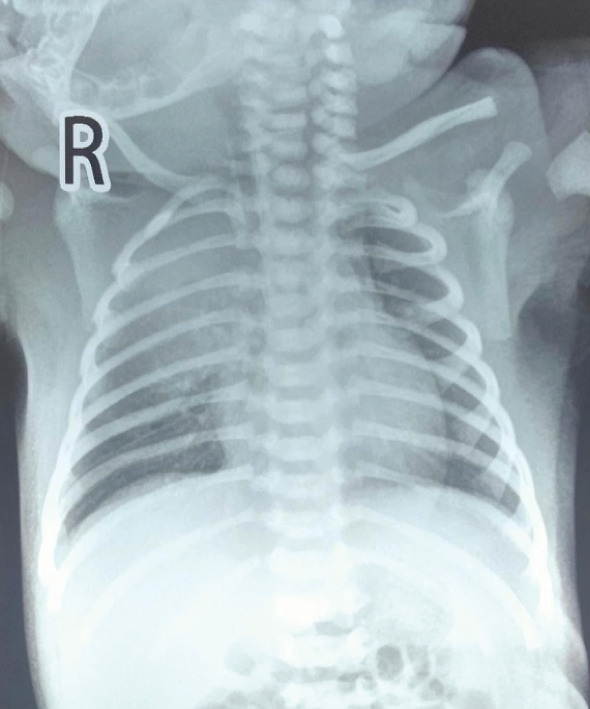
Babygram results.

The patient was then terminated with CS at the tertiary hospital. A male baby weighing 3075 g was born with CS with an AS (Apgar score) of 7‐8‐9. At birth, the baby was then evaluated by neonatology and a babygram (x‐ray) was performed and the results showed suspicion of CPAM type III (Figure 2). The baby was then consulted to the Pediatric Surgery department and advised for CT scan. The baby was discharged and planned for a CT scan during outpatient visit. CT scan at 16 days old showed right intrapulmonary cystic lesion with air fluid level size 33 x 25 x 40 mm in the anterior segment, with the impression of infected type I CCAM.

**Figure 3:**
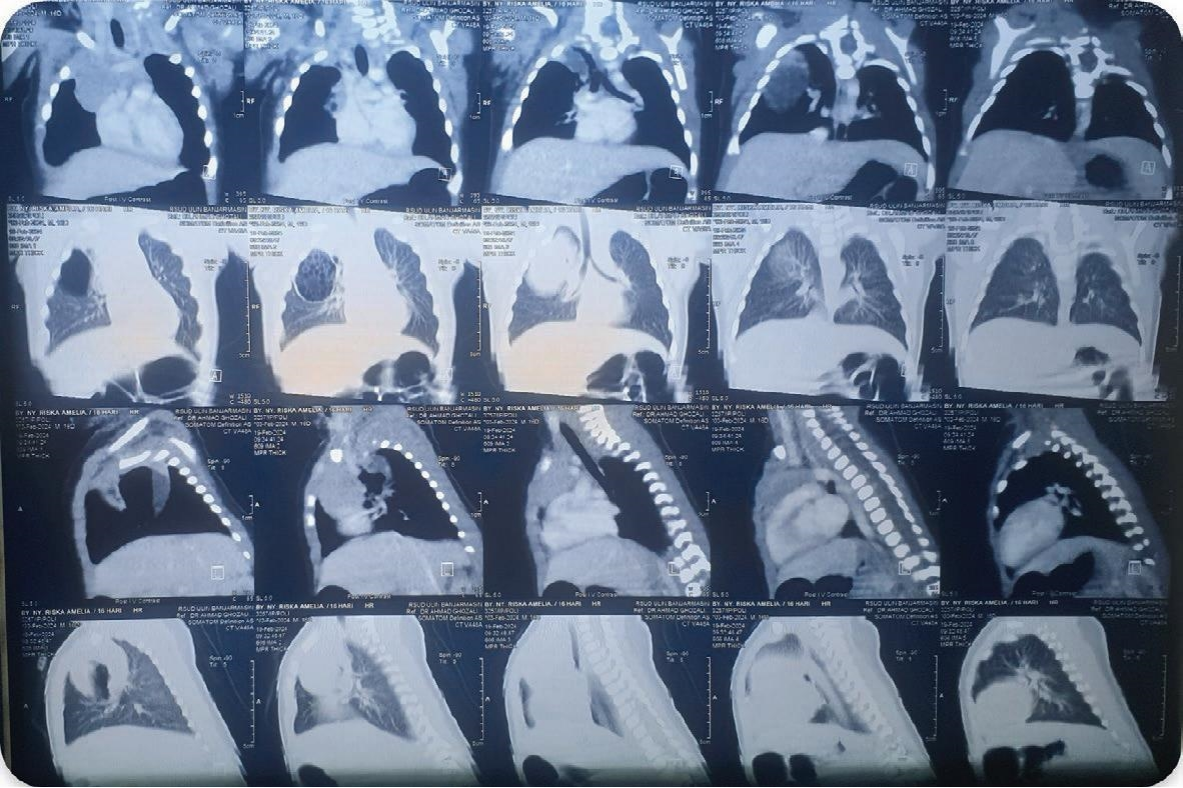
Mediastinal window of CT scan.

The mediastinal window on the CT scan demonstrated midline trachea, main left and right bronchial stem were opened, no visible mediastinal mass or lymph node hypertrophy, cardiovascular within normal limits, and no visible pleural effusion/pericardial effusion (Figure 3).

**Figure 4:**
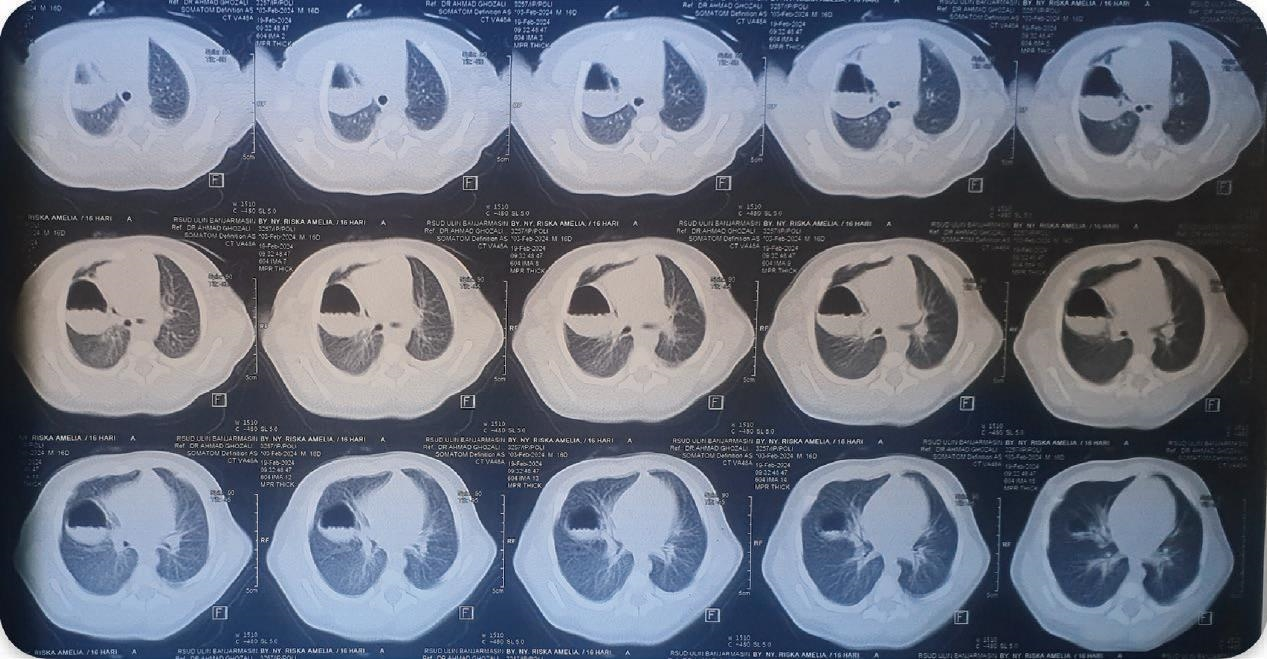
Pulmonary window of CT scan.

**Figure 5:**
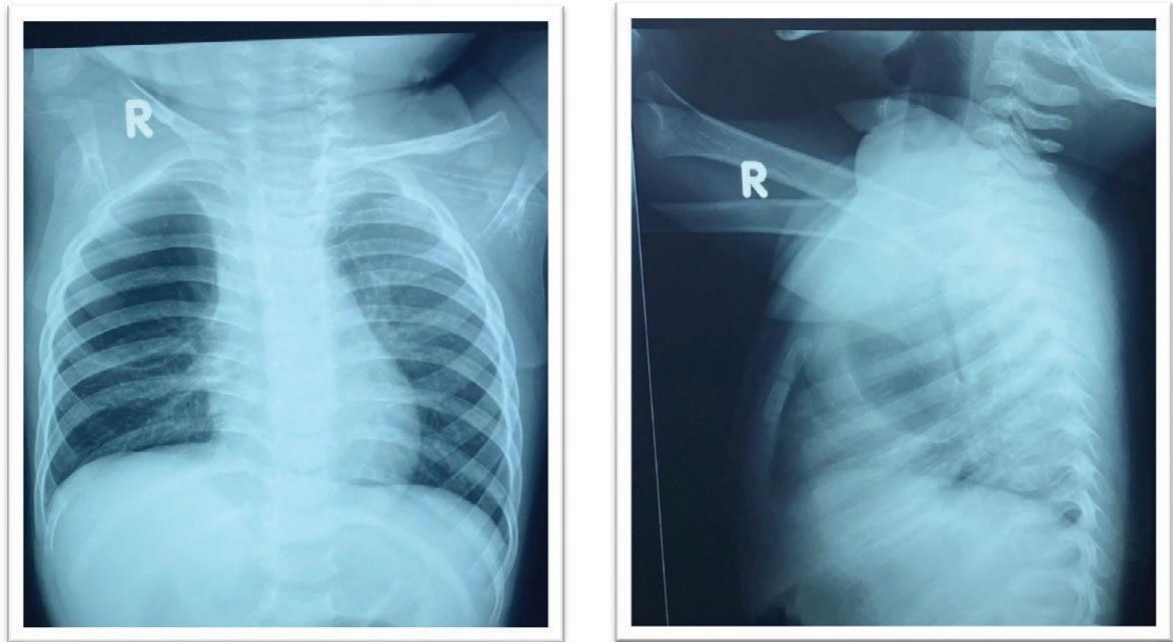
Pulmonary cyst on the superior pulmonary lobe.

On pulmonary window, there were no visible consolidations, infiltrate, ground glass appearance, or fibrosis, and no visible intrapulmonary nodule (Figure 4). The baby was observed through home visit, and at 1 year of age, there were no signs of respiratory distress, and the baby was allowed to have a routine outpatient visit for nonsurgical intervention. Repeat chest x‐ray at 1 year of age revealed a lucent lesion with a thin wall on the superior pulmonary lobe approximately 6 cm consistent with a pulmonary cyst on the superior pulmonary lobe and no cardiomegaly (Figure 5).

## 3. Discussion

Cesarean section was performed with indications of breech presentation in the fetus accompanied by fetal abnormalities of CCAM. This is in accordance with the literature which states that in fetuses with breech abnormalities, it is one of the indications for CS delivery [5].

Congenital fetal abnormality CCAM can be detected during routine antenatal ultrasonography examination. This is in line with the literature stating that the diagnosis is made by imaging [6]. A small portion of lesions detected by fetal ultrasonography will resolve during pregnancy, but most lesions that appear at birth will persist. The widespread use of antenatal ultrasound examination has improved the diagnosis of prenatal CCAM [1]. Detailed family history should be obtained to determine the presence of malignancy and cystic lesions that may be familial such as pleuropulmonary blastoma syndrome (PPB). This includes renal cystic disease, small bowel polyps, cancer or dysplasia in childhood, and a history of spontaneous pneumothorax [1, 7]. Data from large population registrations show that the incidence of congenital lung cysts ranges from 1 per 8300 to 35,000 live births. The large cyst subtype accounts for about 70% of CCAM, or 2–8 per 100,000 live births [2].

CCAM results from abnormalities in the morphogenesis of the lung tree. The molecular mechanisms leading to CCAM formation are unknown but may involve an imbalance between cell proliferation and apoptosis during organogenesis. Disruption of the HOXB5 (homeobox B5) gene has been implicated in this process [2]. This process may be mediated in part by glial cell‐derived neurotrophic factor (GDNF), a growth factor that is highly expressed in organs whose development is characterized by epithelial–mesenchymal interactions. In one report, GDNF was detected in epithelial and endothelial cells from normal fetal lung and epithelial cells from CCAM, whereas it was absent from normal lung tissue obtained from infants and older children (ages 4 months–3 years) [1]. One study found that CCAM resected from fetuses and newborns had twice as much cell proliferation and five times as many apoptotic bodies as normal fetal and neonatal lung tissue [4].

In pregnant women with a predisposition and diagnosis of fetal CCAM, assessment of associated abnormalities and serial ultrasound examinations to monitor changes in CCAM and the development of hydrops are included. Fetuses with large CCAM and/or hydrops have a poor prognosis. In this case, treatment options include antenatal corticosteroids, drainage procedures, fetal surgery, or early delivery [1, 4]. When CCAM is diagnosed prenatally, it can be managed prenatally if there is a risk of fetal hydrops. If the physician identifies this risk in the prenatal period, interventions such as fetal surgery, corticosteroids, or drainage can be performed to prevent fetal death [8].

In the postnatal period, if the infant is symptomatic for respiratory distress, surgical resection is the treatment of choice. In addition, surgical resection is recommended for infants with lesions occupying more than 20% of the hemithorax, bilateral or multifocal cysts, pneumothorax, or a family history of pleuropulmonary blastoma. Often in older children with mild symptoms, resection is also performed to prevent recurrent infection or potential malignancy, especially if the lesion is known to be a Type 4 lesion. For asymptomatic patients, there is debate as to whether patients should undergo elective resection of the lesion or take a conservative approach and observe symptoms [8].

According to Stoker Classification, we established Type I CCAM with bronchial‐bronchiolar origin. This is in accordance with the literature which states that a CT scan is performed at the age of 1 month if the baby has no symptoms, but if the baby has respiratory complaints at birth, a CT scan will be performed immediately on the first day. Surgical procedures are reserved for babies with respiratory symptoms; if respiratory symptoms are found during monitoring, the patient can be planned for surgical resection or drainage. Conservative management depends solely on the basis of respiratory symptoms or size of CCAM. Prognosis is also predicted based on CVR during ultrasound examination, and a previous study has proposed an algorithm whether surgical intervention is needed in fetus with CVR > 1.6 as risk of developing fetal hydrops is increased [9, 10].

In a retrospective series, the clinical profile and outcomes of antenatally diagnosed CCAM were evaluated across 10 cases. The gestational age at diagnosis ranged from 18 to 30 weeks, with CCAM volume ratios (CVR) varying from 0.3 to 2.1. Notably, a CVR > 1.6—traditionally associated with an increased risk of hydrops—was observed in four cases, yet only one of these showed mediastinal shift without associated hydrops or cardiovascular compromise. Most lesions were localized to either the upper or lower lobes and described as cystic, with no associated structural anomalies identified in nine of the 10 cases. Fetal echocardiography was uniformly normal, underscoring the isolated nature of these lesions in the majority of cases. Perinatal outcomes were favorable in most instances, with six cases culminating in full‐term normal vaginal delivery and postnatal conservative management. However, two cases resulted in medical termination of pregnancy, possibly due to lesion size, high CVR, or associated mediastinal shift. Only one neonate required neonatal intensive care unit (NICU) admission due to respiratory distress, reflecting that even sizeable or bilateral lesions may have favorable postnatal outcomes if carefully monitored. This study reinforces the heterogeneity of CCAM presentation and emphasizes the prognostic value of CVR, while also supporting a conservative approach in the absence of hydrops [11].

Active surveillance needs of serial antenatal ultrasonography to assess lesion size, mediastinal shift, and signs of fetal hydrops, and also deteriorating signs needing prenatal intervention. Cavoretto et al. [12] described the prenatal diagnosis and outcomes of echogenic fetal lung lesions and highlighted that some lesions decrease in size or even appear to resolve during pregnancy, although postnatal persistence may still be demonstrated. In our case, the lesion was small (CVR 0.26), no hydrops was identified, and there was no evidence of fetal cardiovascular compromise, since the pregnancy already reached full term then delivery was planned. No antenatal corticosteroids, thoracoamniotic shunting, fetal surgery, or other specific prenatal treatment were performed because there was no clinical indication for fetal intervention [1, 4, 8, 12].

The discrepancy between the early postnatal babygram, which suggested CPAM type III, and the later CT scan, which favored Type I, reflects the limitations of plain radiography in neonatal congenital lung lesions and the dynamic appearance of cystic lesions immediately after birth. Type III lesions are classically microcystic and may appear relatively solid on radiography, whereas Type I lesions are characterized by larger cysts that are more accurately delineated by CT. In the early neonatal period, retained fetal lung fluid, partial aeration, and superimposed infection or air–fluid levels may obscure the true cyst architecture on chest radiograph, making a macrocystic lesion appear more homogeneous. Thus, the CT findings at 16 days of age were considered more reliable for subtype characterization in this case, and the lesion was ultimately interpreted as Type I CCAM [3, 9, 13].

## 4. Conclusion

Cesarean section was performed based on consideration of the mother′s condition with a breech fetus and congenital abnormalities in the form of CCAM obtained from an ultrasound examination. The infant remained clinically stable after birth and throughout serial outpatient follow‐up. No respiratory distress, feeding difficulty, or other major complications were reported during the first year of life. At 1 year of age, chest radiography still showed a pulmonary cyst; the infant remained asymptomatic, and conservative follow‐up without surgery was continued.

## Author Contributions

**Bambang Abimanyu:** conceptualization, investigation, data curation, writing – original draft. **Muhammad Robyanoor Ahyadi Radaam:** investigation, data curation, writing – review & editing. **Ruth Widhiati Raharjo Putri:** investigation, resources, writing – review & editing. **Yosef Dwi Cahyadi Salan:** formal analysis, visualization, writing – review & editing. **Hermin Sabarudin:** supervision, validation, writing – review & editing. **Adek Yeary Wardhani:** methodology, validation, writing – review & editing. **Yakob Togar:** conceptualization, supervision, project administration, writing – review & editing, correspondence.

## Funding

No funding was received for this manuscript.

## Consent

Written informed consent for the publication of the case report and any associated were obtained from the patient prior to submission. The study participant has given consent to participate as well as consent to publish the data.

## Conflicts of Interest

The authors declare no conflicts of interest.

## Data Availability

All data underlying the results are available on request from the authors.
